# 2-[(*E*)-(Naphthalen-2-yl)imino­meth­yl]phenol

**DOI:** 10.1107/S1600536812033843

**Published:** 2012-08-04

**Authors:** Hafiz Muhammad Adeel Sharif, Dildar Ahmed Alvi, S Yousuf

**Affiliations:** a Department of Chemistry, Forman Christian College (A Chartered University), Lahore, Pakistan; b H.E.J. Research Institute of Chemistry, International Center for Chemical and Biological Sciences, University of Karachi, Karachi 75270, Pakistan

## Abstract

In the title compound, C_17_H_13_NO, the azomethine double bond adopts an *E* conformation. The naphthyl ring system and the benzene ring form a dihedral angle of 8.09 (10)°. The near-planar conformation of the molecule is consolidated by an intra­molecular O—H⋯N hydrogen bond, which forms an *S*(6) ring. In the crystal, mol­ecules are arranged in a zigzag fashion parallel to the *c* axis.

## Related literature

For the biological activity of Schiff bases, see: Khan *et al.* (2009 ▶). For the crystal structure of a closely related Schiff base, see: Aslam *et al.* (2012 ▶).
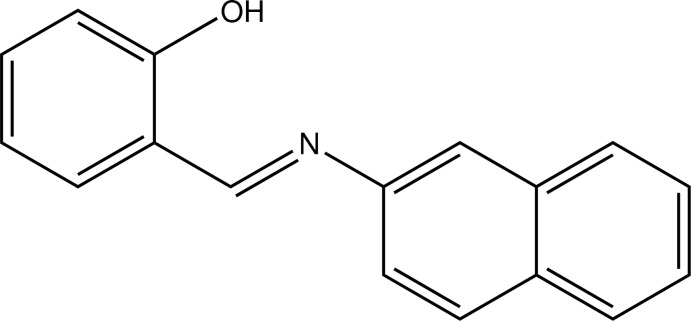


## Experimental

### 

#### Crystal data

C_17_H_13_NO
*M* *_r_* = 247.28
Orthorhombic, 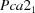

*a* = 13.6348 (17) Å
*b* = 5.8768 (7) Å
*c* = 15.869 (2) Å
*V* = 1271.5 (3) Å^3^
*Z* = 4
Mo *K*α radiation
μ = 0.08 mm^−1^
*T* = 273 K
0.15 × 0.13 × 0.10 mm

#### Data collection

Bruker SMART APEX CCD area-detector diffractometer
Absorption correction: multi-scan (*SADABS*; Bruker, 2000 ▶) *T*_min_ = 0.988, *T*_max_ = 0.992
6852 measured reflections
2300 independent reflections
1655 reflections with *I* > 2σ(*I*)
*R*_int_ = 0.031

#### Refinement

*R*[*F* ^2^ > 2σ(*F* ^2^)] = 0.038
*wR*(*F* ^2^) = 0.089
*S* = 1.00
2300 reflections
176 parameters
2 restraints
H atoms treated by a mixture of independent and constrained refinement
Δρ_max_ = 0.08 e Å^−3^
Δρ_min_ = −0.09 e Å^−3^

### 

Data collection: *SMART* (Bruker, 2000 ▶); cell refinement: *SAINT* (Bruker, 2000 ▶); data reduction: *SAINT*; program(s) used to solve structure: *SHELXS97* (Sheldrick, 2008 ▶); program(s) used to refine structure: *SHELXL97* (Sheldrick, 2008 ▶); molecular graphics: *SHELXTL* (Sheldrick, 2008 ▶); software used to prepare material for publication: *SHELXTL*, *PARST* (Nardelli, 1995 ▶) and *PLATON* (Spek, 2009 ▶).

## Supplementary Material

Crystal structure: contains datablock(s) global, I. DOI: 10.1107/S1600536812033843/pv2571sup1.cif

Structure factors: contains datablock(s) I. DOI: 10.1107/S1600536812033843/pv2571Isup2.hkl

Supplementary material file. DOI: 10.1107/S1600536812033843/pv2571Isup3.cml

Additional supplementary materials:  crystallographic information; 3D view; checkCIF report

## Figures and Tables

**Table 1:** Hydrogen-bond geometry (Å, °)

*D*—H⋯*A*	*D*—H	H⋯*A*	*D*⋯*A*	*D*—H⋯*A*
O1—H1*C*⋯N1	0.86 (2)	1.86 (2)	2.623 (3)	147 (2)

**Fig. 1.**
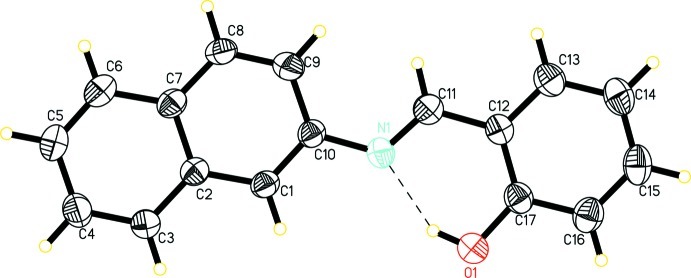
The molecular structure of the title compound with the atom numbering scheme. Displacement ellipsoids are drawn at the 30% probability level. H atoms are presented as small spheres of arbitrary radius.

**Fig. 2.**
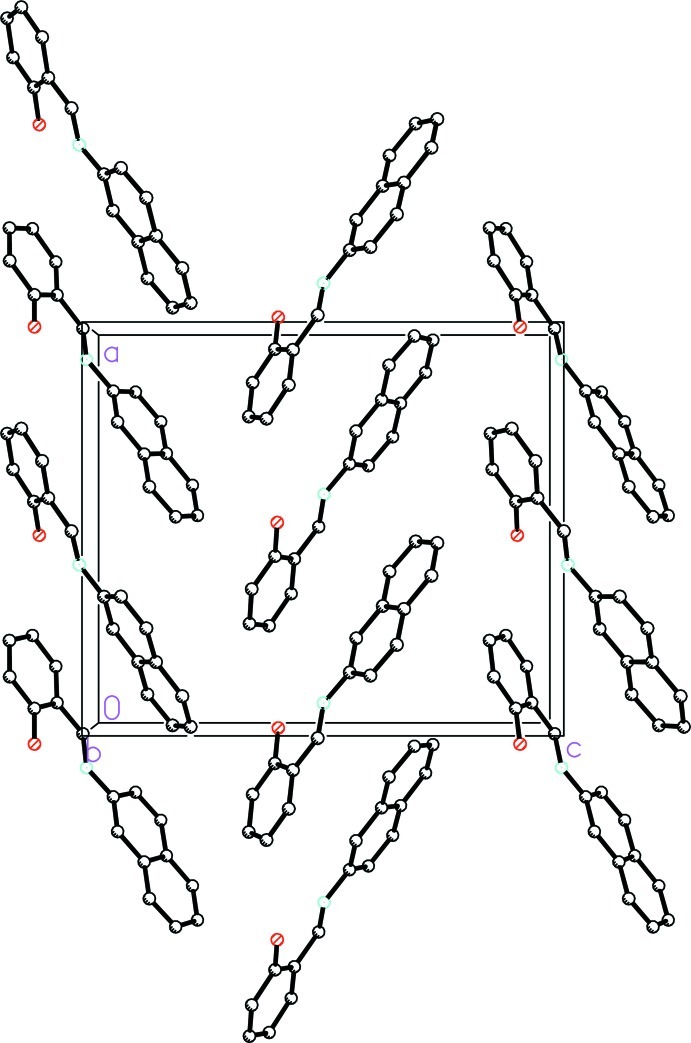
A view of the unit cell of the title compound showing molecular packing. H atoms were omitted for clarity.

## supplementary crystallographic information

### Comment

Schiff bases represent a broad class of organic compounds that are reported to have a wide range of biological activities (Khan *et al.*, 2009). The title compound was synthesized as a part of our ongoing research to study the biological activities of structurally diverse Schiff bases. The title compound (Fig. 1) is composed of a naphthyl (C1–C10) and a benzene rings (C12–C17) linked through an azomethine (C═N = 1.275 (2) Å) double bond which adopts an *E* configuration. The dihedral angle between the naphthyl and the benzene rings is 8.09 (10)° with maximum deviation of 0.013 (3) Å for C5 atom from the root mean square plane of the naphthyl ring. The bond lengths and angle in the title molecule are similar to the corresponding bond lengths and angles in a closely related Schiff base (Aslam *et al.* 2012). The molecular structure is stabilized by an intramolecular O1—H1C···N1 hydrogen bond to form *S*(6) graph set ring motif. In the crystal structure the molecules are arranged in a zig zag fashion to form sheets parallel to the *c*-axis (Fig.2).

### Experimental

4-Chloroaniline (1 ml, 7.29 mmol) was dissolved in analytical grade methanol (10 ml) by continuous stirring followed by the addition of sSalicylaldehyde (0.76 ml, 0.7 mmol) and glacial acetic acid (0.5 ml). The reaction mixture was refluxed at 330–353 K on a hot plate for 2 h with continuous stirring. The progress of the reaction was monitored by TLC. On the completion of the reaction, the product was obtained as dark orange precipitates, which were filtered, washed with distilled water and dried to obtained 1.43 g (77% yield) title compound. The product was dissolved and slow evaporation of a methanol solution affording light yellow crystals suitable for single-crystal X-ray diffraction studies. All chemicals were purchased from Sigma-Aldrich.

### Refinement

H atoms on carbon atoms were positioned geometrically with C—H = 0.93 Å, and constrained to ride on their parent atoms with *U*_iso_(H)= 1.2*U*_eq_(C). The H atoms on the oxygen (O–H = 0.858 (10) Å) was located in difference Fourier maps and refined isotropically. Due to lack of sufficient anamolous effects, an absolute structure was not determined and the Friedle pairs (1082) were not merged.

### Crystal data

**Table:**

C_17_H_13_NO	*F*(000) = 520
*M**_r_* = 247.28	*D*_x_ = 1.292 Mg m^−^^3^
Orthorhombic, *P**c**a*2_1_	Mo *K*α radiation, λ = 0.71073 Å
Hall symbol: P 2c -2ac	Cell parameters from 1294 reflections
*a* = 13.6348 (17) Å	θ = 2.6–27.8°
*b* = 5.8768 (7) Å	µ = 0.08 mm^−^^1^
*c* = 15.869 (2) Å	*T* = 273 K
*V* = 1271.5 (3) Å^3^	Block, yellow
*Z* = 4	0.15 × 0.13 × 0.10 mm

### Data collection

**Table:**

Bruker SMART APEX CCD area-detector diffractometer	2300 independent reflections
Radiation source: fine-focus sealed tube	1655 reflections with *I* > 2σ(*I*)
Graphite monochromator	*R*_int_ = 0.031
ω scan	θ_max_ = 25.5°, θ_min_ = 2.6°
Absorption correction: multi-scan (*SADABS*; Bruker, 2000)	*h* = −16→16
*T*_min_ = 0.988, *T*_max_ = 0.992	*k* = −7→6
6852 measured reflections	*l* = −19→19

### Refinement

**Table:**

Refinement on *F*^2^	Primary atom site location: structure-invariant direct methods
Least-squares matrix: full	Secondary atom site location: difference Fourier map
*R*[*F*^2^ > 2σ(*F*^2^)] = 0.038	Hydrogen site location: inferred from neighbouring sites
*wR*(*F*^2^) = 0.089	H atoms treated by a mixture of independent and constrained refinement
*S* = 1.00	*w* = 1/[σ^2^(*F*_o_^2^) + (0.0383*P*)^2^] where *P* = (*F*_o_^2^ + 2*F*_c_^2^)/3
2300 reflections	(Δ/σ)_max_ < 0.001
176 parameters	Δρ_max_ = 0.08 e Å^−^^3^
2 restraints	Δρ_min_ = −0.09 e Å^−^^3^

### Special details

**Table:**

Geometry. All e.s.d.'s (except the e.s.d. in the dihedral angle between two l.s. planes) are estimated using the full covariance matrix. The cell e.s.d.'s are taken into account individually in the estimation of e.s.d.'s in distances, angles and torsion angles; correlations between e.s.d.'s in cell parameters are only used when they are defined by crystal symmetry. An approximate (isotropic) treatment of cell e.s.d.'s is used for estimating e.s.d.'s involving l.s. planes.
Refinement. Refinement of *F*^2^ against ALL reflections. The weighted *R*-factor *wR* and goodness of fit *S* are based on *F*^2^, conventional *R*-factors *R* are based on *F*, with *F* set to zero for negative *F*^2^. The threshold expression of *F*^2^ > σ(*F*^2^) is used only for calculating *R*-factors(gt) *etc*. and is not relevant to the choice of reflections for refinement. *R*-factors based on *F*^2^ are statistically about twice as large as those based on *F*, and *R*- factors based on ALL data will be even larger.

### Fractional atomic coordinates and isotropic or equivalent isotropic displacement parameters (Å^2^)

**Table:**

	*x*	*y*	*z*	*U*_iso_*/*U*_eq_	
O1	0.51540 (13)	1.1177 (3)	0.40612 (13)	0.0846 (6)	
N1	0.58509 (13)	0.7914 (3)	0.50171 (12)	0.0592 (5)	
C1	0.73882 (15)	0.8452 (4)	0.56875 (14)	0.0542 (5)	
H1B	0.7413	0.9851	0.5415	0.065*	
C2	0.81650 (15)	0.7835 (4)	0.62265 (13)	0.0519 (5)	
C3	0.89755 (15)	0.9265 (4)	0.63756 (14)	0.0626 (6)	
H3A	0.9012	1.0662	0.6103	0.075*	
C4	0.97028 (19)	0.8641 (4)	0.69100 (17)	0.0718 (7)	
H4A	1.0229	0.9616	0.7001	0.086*	
C5	0.96686 (19)	0.6547 (5)	0.73249 (15)	0.0707 (7)	
H5A	1.0167	0.6140	0.7695	0.085*	
C6	0.89085 (18)	0.5111 (4)	0.71880 (14)	0.0664 (7)	
H6A	0.8901	0.3702	0.7455	0.080*	
C7	0.81231 (16)	0.5715 (4)	0.66451 (13)	0.0552 (6)	
C8	0.73097 (17)	0.4295 (4)	0.64880 (16)	0.0662 (7)	
H8A	0.7274	0.2883	0.6750	0.079*	
C9	0.65784 (16)	0.4950 (4)	0.59615 (17)	0.0694 (7)	
H9A	0.6051	0.3977	0.5869	0.083*	
C10	0.66026 (15)	0.7071 (3)	0.55524 (14)	0.0527 (5)	
C11	0.50538 (16)	0.6824 (4)	0.49001 (14)	0.0591 (6)	
H11A	0.4976	0.5400	0.5147	0.071*	
C12	0.42625 (15)	0.7755 (4)	0.43905 (13)	0.0538 (6)	
C13	0.33980 (16)	0.6545 (4)	0.43146 (16)	0.0676 (6)	
H13A	0.3340	0.5137	0.4577	0.081*	
C14	0.26177 (19)	0.7392 (5)	0.38553 (15)	0.0753 (7)	
H14A	0.2040	0.6559	0.3808	0.090*	
C15	0.27038 (18)	0.9489 (5)	0.34667 (16)	0.0738 (7)	
H15A	0.2179	1.0067	0.3159	0.089*	
C16	0.35529 (17)	1.0725 (5)	0.35294 (16)	0.0709 (7)	
H16A	0.3606	1.2121	0.3257	0.085*	
C17	0.43405 (15)	0.9890 (4)	0.40022 (15)	0.0596 (6)	
H1C	0.5580 (15)	1.045 (4)	0.4354 (15)	0.085 (9)*	

### Atomic displacement parameters (Å^2^)

**Table:**

	*U*^11^	*U*^22^	*U*^33^	*U*^12^	*U*^13^	*U*^23^
O1	0.0667 (11)	0.0733 (12)	0.1138 (16)	−0.0103 (10)	−0.0150 (11)	0.0200 (12)
N1	0.0481 (10)	0.0643 (12)	0.0652 (12)	0.0001 (9)	−0.0006 (9)	0.0003 (10)
C1	0.0549 (12)	0.0528 (13)	0.0549 (13)	0.0019 (10)	0.0028 (11)	0.0091 (11)
C2	0.0526 (12)	0.0541 (13)	0.0490 (13)	0.0030 (10)	0.0050 (10)	0.0028 (12)
C3	0.0625 (14)	0.0623 (15)	0.0628 (15)	−0.0067 (12)	−0.0046 (12)	0.0083 (13)
C4	0.0656 (16)	0.080 (2)	0.0693 (15)	−0.0074 (13)	−0.0103 (14)	−0.0022 (15)
C5	0.0682 (16)	0.0849 (19)	0.0589 (14)	0.0102 (14)	−0.0107 (12)	0.0035 (15)
C6	0.0729 (17)	0.0688 (15)	0.0576 (16)	0.0126 (14)	0.0048 (14)	0.0094 (13)
C7	0.0581 (13)	0.0592 (14)	0.0484 (13)	0.0059 (11)	0.0069 (11)	0.0063 (12)
C8	0.0656 (16)	0.0570 (14)	0.0759 (17)	−0.0038 (12)	0.0015 (14)	0.0200 (13)
C9	0.0588 (14)	0.0648 (16)	0.0847 (17)	−0.0092 (12)	0.0021 (14)	0.0113 (15)
C10	0.0483 (12)	0.0548 (14)	0.0551 (13)	0.0041 (10)	0.0036 (12)	0.0051 (12)
C11	0.0591 (14)	0.0567 (13)	0.0616 (15)	0.0047 (11)	0.0037 (12)	−0.0055 (12)
C12	0.0506 (12)	0.0585 (14)	0.0523 (13)	0.0013 (11)	0.0029 (10)	−0.0084 (12)
C13	0.0669 (14)	0.0712 (16)	0.0649 (15)	−0.0051 (13)	−0.0020 (13)	−0.0112 (14)
C14	0.0649 (15)	0.095 (2)	0.0659 (17)	−0.0086 (14)	−0.0118 (13)	−0.0116 (16)
C15	0.0617 (15)	0.096 (2)	0.0642 (16)	0.0132 (14)	−0.0109 (13)	−0.0180 (16)
C16	0.0726 (16)	0.0752 (18)	0.0649 (16)	0.0103 (14)	−0.0071 (15)	−0.0064 (14)
C17	0.0521 (13)	0.0641 (15)	0.0627 (15)	0.0007 (11)	0.0031 (12)	−0.0067 (13)

### Geometric parameters (Å, º)

**Table:**

O1—C17	1.346 (3)	C7—C8	1.410 (3)
O1—H1C	0.86 (2)	C8—C9	1.356 (3)
N1—C11	1.275 (2)	C8—H8A	0.9300
N1—C10	1.421 (2)	C9—C10	1.406 (3)
C1—C10	1.361 (3)	C9—H9A	0.9300
C1—C2	1.409 (3)	C11—C12	1.455 (3)
C1—H1B	0.9300	C11—H11A	0.9300
C2—C3	1.408 (3)	C12—C13	1.382 (3)
C2—C7	1.413 (3)	C12—C17	1.402 (3)
C3—C4	1.355 (3)	C13—C14	1.382 (3)
C3—H3A	0.9300	C13—H13A	0.9300
C4—C5	1.396 (3)	C14—C15	1.383 (3)
C4—H4A	0.9300	C14—H14A	0.9300
C5—C6	1.354 (3)	C15—C16	1.370 (3)
C5—H5A	0.9300	C15—H15A	0.9300
C6—C7	1.419 (3)	C16—C17	1.399 (3)
C6—H6A	0.9300	C16—H16A	0.9300
			
C17—O1—H1C	108.4 (18)	C8—C9—H9A	119.4
C11—N1—C10	121.78 (19)	C10—C9—H9A	119.4
C10—C1—C2	122.3 (2)	C1—C10—C9	118.3 (2)
C10—C1—H1B	118.9	C1—C10—N1	116.99 (18)
C2—C1—H1B	118.9	C9—C10—N1	124.66 (19)
C3—C2—C1	122.6 (2)	N1—C11—C12	121.6 (2)
C3—C2—C7	118.60 (19)	N1—C11—H11A	119.2
C1—C2—C7	118.80 (19)	C12—C11—H11A	119.2
C4—C3—C2	121.2 (2)	C13—C12—C17	119.1 (2)
C4—C3—H3A	119.4	C13—C12—C11	119.2 (2)
C2—C3—H3A	119.4	C17—C12—C11	121.6 (2)
C3—C4—C5	120.6 (2)	C12—C13—C14	121.2 (2)
C3—C4—H4A	119.7	C12—C13—H13A	119.4
C5—C4—H4A	119.7	C14—C13—H13A	119.4
C6—C5—C4	119.9 (2)	C13—C14—C15	119.4 (2)
C6—C5—H5A	120.0	C13—C14—H14A	120.3
C4—C5—H5A	120.0	C15—C14—H14A	120.3
C5—C6—C7	121.3 (2)	C16—C15—C14	120.8 (2)
C5—C6—H6A	119.4	C16—C15—H15A	119.6
C7—C6—H6A	119.4	C14—C15—H15A	119.6
C8—C7—C2	118.1 (2)	C15—C16—C17	120.1 (3)
C8—C7—C6	123.5 (2)	C15—C16—H16A	120.0
C2—C7—C6	118.4 (2)	C17—C16—H16A	120.0
C9—C8—C7	121.3 (2)	O1—C17—C16	118.2 (2)
C9—C8—H8A	119.4	O1—C17—C12	122.3 (2)
C7—C8—H8A	119.4	C16—C17—C12	119.4 (2)
C8—C9—C10	121.2 (2)		
			
C10—C1—C2—C3	−179.4 (2)	C8—C9—C10—C1	0.9 (3)
C10—C1—C2—C7	−0.4 (3)	C8—C9—C10—N1	−177.6 (2)
C1—C2—C3—C4	178.8 (2)	C11—N1—C10—C1	−174.8 (2)
C7—C2—C3—C4	−0.2 (3)	C11—N1—C10—C9	3.8 (3)
C2—C3—C4—C5	0.3 (4)	C10—N1—C11—C12	176.38 (18)
C3—C4—C5—C6	0.7 (4)	N1—C11—C12—C13	−177.2 (2)
C4—C5—C6—C7	−1.8 (4)	N1—C11—C12—C17	0.2 (3)
C3—C2—C7—C8	−179.76 (19)	C17—C12—C13—C14	0.8 (3)
C1—C2—C7—C8	1.2 (3)	C11—C12—C13—C14	178.27 (19)
C3—C2—C7—C6	−0.9 (3)	C12—C13—C14—C15	−0.1 (3)
C1—C2—C7—C6	−179.89 (19)	C13—C14—C15—C16	0.3 (4)
C5—C6—C7—C8	−179.3 (2)	C14—C15—C16—C17	−1.1 (4)
C5—C6—C7—C2	1.9 (3)	C15—C16—C17—O1	−178.7 (2)
C2—C7—C8—C9	−1.0 (3)	C15—C16—C17—C12	1.7 (3)
C6—C7—C8—C9	−179.8 (2)	C13—C12—C17—O1	178.9 (2)
C7—C8—C9—C10	−0.1 (4)	C11—C12—C17—O1	1.5 (3)
C2—C1—C10—C9	−0.7 (3)	C13—C12—C17—C16	−1.6 (3)
C2—C1—C10—N1	177.97 (18)	C11—C12—C17—C16	−179.0 (2)

### Hydrogen-bond geometry (Å, º)

**Table:**

*D*—H···*A*	*D*—H	H···*A*	*D*···*A*	*D*—H···*A*
O1—H1*C*···N1	0.86 (2)	1.86 (2)	2.623 (3)	147 (2)
